# Dysregulation of circulating damage-associated molecular patterns in diabetic foot syndrome

**DOI:** 10.3389/fimmu.2026.1849387

**Published:** 2026-06-22

**Authors:** Elena Uyy, Viorel-Iulian Suica, Luminita Ivan, Raluca Maria Boteanu, Diana Valentina Uta, Elena Georgiana Bernea, Dragoş Eugen Georgescu, Ovidiu Chiriac, Maya Simionescu, Felicia Antohe

**Affiliations:** 1 Proteomics Department, Institute of Cellular Biology and Pathology “Nicolae Simionescu”, of Romanian Academy, Bucharest, Romania; 2 Department of General Surgery, “Dr. Ion Cantacuzino” Clinical Hospital, Bucharest, Romania

**Keywords:** acute-phase proteins, DAMPs, diabetic foot syndrome, LC-MS/MS, toll-like receptor 4, vascular inflammation

## Abstract

**Background:**

Diabetic foot syndrome (DFS) is characterized by chronic inflammation, thrombotic imbalance, and impaired wound healing, yet systemic molecular alterations underlying this complication remain incompletely defined. In this study, we combined clinical plasma proteomics with experimental pharmacological modulation to characterize a circulating damage-associated molecular pattern (DAMP)-related signature linked to systemic inflammatory signaling in DFS.

**Methods:**

Plasma samples from patients with type 2 diabetes with and without DFS, including individuals with varying degrees of limb ischemia, were analyzed using liquid chromatography-tandem mass spectrometry. Differentially abundant proteins were evaluated in relation to inflammatory, hematological, and metabolic parameters. Pharmacological responsiveness was assessed in a murine diabetic ischemic wound model treated with a selective Toll-like receptor 4 (TLR4) inhibitor.

**Results:**

Proteomic analysis identified coordinated differences in the abundance of multiple acute-phase and stress-associated proteins, including serum amyloid A1, serum amyloid A2, serum amyloid P component, S100A8, defensin alpha 1B, fibrinogen chains, heat shock protein family A member 5, thymosin beta 4, fibronectin 1, and tenascins. These proteins exhibited differences between DFS patients and diabetic controls and were explored in relation to systemic inflammatory variables. Several DAMPs demonstrated reproducible patterns across the studied groups, suggesting the presence of a coordinated circulating molecular pattern rather than isolated changes in individual proteins. In diabetic ischemic mice, TLR4 inhibition was associated with altered abundance of several circulating proteins, including reductions in selected amyloid-associated proteins. These observations suggest an association between modulation of innate immune signaling pathways and circulating protein profiles.

**Conclusion:**

Overall, these findings support a systemic alteration in circulating DAMP abundance in DFS and provide exploratory clinical and experimental evidence to guide future investigations into DAMP-mediated inflammatory pathways in diabetic ischemic complications. Proteomics data are available via ProteomeXchange with identifier PXD073507.

## 1 Introduction

Diabetic Foot Syndrome (DFS) is a major and multifactorial complication of diabetes mellitus, characterized by ischemic tissue damage, ulceration, and secondary infection, that may progress to amputation if inadequately managed. Despite advances in diabetes care, individuals with DFS continue to have markedly higher amputation rates compared with those without diabetes (1). Therapeutic options for promoting ulcer healing in patients who are not candidates for revascularization remain limited and largely unsupported by strong evidence (2).

Current international guidelines enable clinicians to stratify patients according to disease severity, tailor therapeutic interventions, and facilitate communication across multidisciplinary teams. This approach improves the comparability of clinical studies and supports the development of evidence-based management strategies (3).

Peripheral ischemia, neuropathy, and impaired immune responses are key contributors to the development and progression of DFS, delaying wound healing and increasing the risk of severe complications (2, 4, 5). The multiple pathological processes underlying DFS underscore the need for early detection, accurate ulcer classification, and targeted therapeutic interventions (6–10).

Endothelial dysfunction, oxidative stress, and alterations in lipid metabolism further contribute to the progression of diabetic foot lesions (11, 12). In addition, diabetic immunopathy, characterized by dysregulated inflammatory responses, further compromises wound healing (13). Elevated inflammatory and hemostatic markers such as C-reactive protein, interleukin 6, D-dimer, fibrinogen, and matrix metalloproteinases reflect disease severity and associated cardiovascular risk (14–16), while the prognostic role of oxidative stress and angiogenesis-related molecules remains to be fully elucidated (17).

Among the molecular mechanisms linking inflammation and vascular injury in DFS, Damage- associated molecular patterns (DAMPs) play a pivotal role. These endogenous molecules, released from stressed or damaged cells, activate immune responses through receptors such as Toll-like receptor 4 (TLR4) (18, 19). Pharmacological targeting of the DAMP-TLR4 axis using a selective inhibitor may modulate the inflammatory response and represents a potential therapeutic approach to prevent DFS progression.

In this study, we assessed clinical, biochemical, and proteomic data from patients with diabetes without foot syndrome (D) and with DFS to characterize systemic DAMP alterations associated with disease severity. To explore mechanistic relevance, a murine model of diabetic ischemia was used to assess the effects of selective TLR4 inhibition on circulating DAMP abundance. Our data suggest that (1): DFS is associated with a systemic alterations in circulating DAMP abundance reflecting heightened inflammatory, thrombotic, and extracellular matrix remodeling stress (2); a subset of these DAMPs discriminates DFS from diabetic patients without foot complications; and (3) pharmacological TLR4 inhibition selectively modulates acute-phase DAMPs, providing functional evidence consistent with TLR4 involvement in systemic inflammatory regulation and highlighting candidate molecules that may warrant further investigation in DFS.

## 2 Materials and methods

### 2.1 Subjects

Patient selection targeted individuals with type 2 diabetes, previously enrolled in the Romanian National Diabetes Control Program. Patients with a history of lower-limb revascularization, vasodilator therapy, psychiatric disorders, or oncological diseases were excluded. A total of 33 patients were therefore included in the study: 9 patients with type 2 diabetes without foot complications (D group) and 24 patients with diabetic foot syndrome (DFS group). All DFS patients were hospitalized for surgical treatment of diabetic foot lesions, following the diagnosis of different stages of ulceration according to the Wagner-Armstrong classification system, based on ulcer depth, infection, and severity of ischemia. Clinical signs of peripheral arteriopathy and critical limb ischemia, such as trophic skin changes, pallor, or delayed wound healing, were also documented. Human blood samples were obtained from “Dr. I. Cantacuzino” Hospital (Bucharest, Romania), in collaboration with the National Institute of Diabetes, Nutrition and Metabolic Diseases “Prof. N.C. Paulescu” (Bucharest, Romania). Due to sample availability and quality control criteria, slightly different sample numbers were used for specific analyses, as indicated in the corresponding figure legends. The study protocol was reviewed and approved by the Ethics Committees of both institutions and by the Institute of Cellular Biology and Pathology “Nicolae Simionescu” (ICBP-NS), approval no. 04/26 July 2022. All procedures were conducted in accordance with the Declaration of Helsinki, and written informed consent was obtained from all participants prior to inclusion.

### 2.2 Blood collection and processing

Venous blood samples were collected from all patients at hospital admission under fasting conditions. Clinical blood parameters measured included uric acid, alanine aminotransferase (ALT), activated partial thromboplastin time (APTT, ratio and seconds), aspartate aminotransferase (AST), basophil count and percentage, total cholesterol, mean corpuscular hemoglobin concentration (MCHC), creatinine, eosinophil count and percentage, fasting glucose, hematocrit (HCT), mean corpuscular hemoglobin (MCH), hemoglobin, international normalized ratio (INR), total white blood cell (WBC) count, lymphocyte count and percentage, monocyte count and percentage, neutrophil count and percentage, red blood cell (RBC) count, platelet distribution width (PDW), serum potassium (K^+^), C-reactive protein (CRP), serum sodium (Na^+^), triglycerides, platelet count (PLT), serum urea, mean corpuscular volume (MCV), and mean platelet volume (MPV) (see **Table 1**).

**Table 1:** Clinical, vascular, infectious, surgical, and biochemical characteristics of patients with diabetes (D) and diabetic foot syndrome (DFS).

Demographic and clinical characteristics of the study cohort		
A. Demographic characteristics		
Variable	D group (n=10)	DFS group (n=24)
Age (years)	63.14 ± 9.77	66.35 ± 9.90
Female sex, n (%)	7 (70.0)	4 (16.7)
B. Vascular status		
Variable	D group	DFS group
Critical limb ischemia, n (%)	0 (0)	12 (50.0)
Arteriopathy, n (%)	0 (0)	18 (75.0)
C. Diabetic foot severity (DFS group only)		
Variable	D group	DFS group
Infection present, n (%)	–	17 (70.8)
Wagner-Armstrong stage 2D, n (%)	–	2 (8.3)
Wagner-Armstrong stage 3D, n (%)	–	11 (45.8)
Wagner-Armstrong stage 4D, n (%)	–	2 (8.3)
Wagner-Armstrong stage 5D, n (%)	–	9 (37.5)
D. Surgical history (DFS group only)		
Variable	D group	DFS group
Previous amputation, n (%)	–	11 (47.8)
Previous debridement, n (%)	–	3 (13.0)
Current surgical treatment, n (%)	–	24 (100)
E. Biochemical parameters		
Variable	D group	DFS group
Uric acid (mg/dL)	5.06 ± 1.61	6.28 ± 3.54
ALT (U/L)	24.22 ± 10.01	21.38 ± 18.84
AST (U/L)	24.88 ± 8.36	17.66 ± 6.95*
Creatinine (mg/dL)	0.80 ± 0.17	1.43 ± 1.11*
Urea (mg/dL)	38.72 ± 6.16	53.51 ± 23.02
Glucose (mg/dL)	161.16 ± 81.08	179.84 ± 100.59
Total cholesterol (mg/dL)	202.05 ± 45.17	139.81 ± 37.41**
Triglycerides (mg/dL)	175.39 ± 31.01	176.99 ± 48.28
Sodium (mmol/L)	140.38 ± 1.80	137.11 ± 3.79
Potassium (mmol/L)	4.66 ± 0.28	4.47 ± 0.70
CRP (mg/dL)	30.65 ± 32.17	126.15 ± 89.33
INR	1.08 ± 0.08	1.14 ± 0.09
F. Hematological parameters		
Variable	D group	DFS group
WBC (×10³/µL)	8.31 ± 3.17	13.48 ± 4.66**
Neutrophils (×10³/µL)	4.80 ± 2.07	10.46 ± 4.47***
Neutrophils (%)	57.34 ± 8.98	73.90 ± 15.41****
Lymphocytes (×10³/µL)	2.62 ± 1.47	1.85 ± 0.40
Lymphocytes (%)	27.35 ± 15.00	16.53 ± 11.22**
Monocytes (×10³/µL)	0.71 ± 0.37	0.96 ± 0.38
Eosinophils (×10³/µL)	0.20 ± 0.11	0.15 ± 0.20
Hemoglobin (g/dL)	12.84 ± 0.62	11.63 ± 1.74*
Hematocrit (%)	42.11 ± 8.62	34.23 ± 5.16***
RBC (×10^6^/µL)	4.71 ± 0.71	3.98 ± 0.63**
MCH (pg)	27.64 ± 3.03	29.36 ± 2.61
MCHC (g/dL)	32.73 ± 1.08	33.99 ± 1.14**
MCV (fL)	84.33 ± 8.52	86.34 ± 6.69
Platelets (×10³/µL)	236.83 ± 28.67	341.42 ± 116.41**
PDW (fL)	15.47 ± 1.62	11.13 ± 3.37**
MPV (fL)	10.81 ± 1.62	9.82 ± 2.32*
G. Coagulation		
Variable	D group	DFS group
APTT (s)	–	36.24 ± 10.45
APTT ratio	–	3.11 ± 8.36
H. Additional indices		
Variable	D group	DFS group
Basophils (%)	0.49 ± 0.25	0.40 ± 0.28
Basophils (×10³/µL)	0.04 ± 0.04	0.05 ± 0.03

Additional blood samples were collected for plasma Western blotting and mass spectrometry analyses. Total protein concentrations were quantified using the BCA Protein Assay Kit (Thermo Scientific, Rockford, IL, USA). Hospital laboratory data were used to compare clinical and biochemical characteristics between D and DFS patients. These results were thereafter correlated with molecular findings.

### 2.3 Murine model of diabetic limb ischemia

Male wild-type Mus musculus, C57BL/6J mice, aged 8–12 weeks, were bred in-house at the animal care facility of ICBP-NS (Bucharest, Romania). Diabetes was induced by five doses of intraperitoneal (ip) streptozotocin (Sigma-Aldrich, St. Louis, MO, USA) at 40 mg/kg^/^day. Mice with blood glucose levels exceeding 240 mg/dL were considered diabetic, and plantar wounds were generated. For a subset of animals, hindlimb ischemia was induced simultaneously with the plantar wounds, 14 days after the first streptozotocin injection, by double ligation and excision of the intermediate femoral artery.

Treatment with TAK-242 (3 mg/kg body weight, ip; Calbiochem/EMD Millipore, USA, Merck KGaA, Darmstadt, Germany) was initiated immediately after ischemia induction and administered every two days for 14 days. Three experimental groups were established: DW (diabetic wounds, n=3), diabetic mice with plantar wounds only; DIW (diabetic ischemic wounds, n=6), diabetic mice with hindlimb ischemia and plantar wounds; and TDIW (treated diabetic ischemic wounds, n=5), diabetic mice with hindlimb ischemia and plantar wounds, treated with the TLR4 inhibitor (TAK-242). Blood was harvested after the treatment and serum isolated for mass spectrometry analyses. Blood glucose levels and body weight were monitored during the experimental protocol to confirm the diabetic phenotype and general metabolic status of the animals.

All animal experiments were conducted according to institutional guidelines and Romanian law and were approved by the Ethics Committee of ICBP-NS under protocol no. 11/10 April 2023. The study followed the Animal Research: Reporting of *In Vivo* Experiments (ARRIVE) guidelines.

### 2.4 Western blot assays

Equal amounts of plasma protein were mixed 1:1 with sample buffer (2% SDS, 0.25 M Tris, 2 mM EDTA, 10% glycerol, 1% 2-mercaptoethanol, 0.5% bromophenol blue). Protein denaturation was performed under reducing conditions at 98 °C for 3 minutes. Samples were loaded onto 12% SDS–polyacrylamide gels, and 7 µl of prestained low-range molecular weight marker was included on each gel. Electrophoresis was performed at 250 V per gel.

Proteins were transferred to nitrocellulose membranes (Bio-Rad Laboratories, Germany) and analyzed by Western blotting, as previously described (20). Membrane transfer efficiency was verified by Ponceau S staining, and non-specific binding was blocked using 5% non-fat dry milk in Tris-buffered saline (TBS) containing 0.05% Tween 20 (pH 7.6).

Membranes were incubated for 2 h with primary antibodies against HSPA5 (Abcam, ab21685, Cambridge, UK), fibronectin (Abcam, ab6328, Cambridge, UK), fibrinogen gamma (Thermo Scientific, MA5-47028, Rockford, IL, USA), S100A8 (Thermo Scientific, PA5-79948, Rockford, IL, USA) and SAA1/SAA2 (Thermo Scientific, PA5-102456, Rockford, IL, USA) diluted in TBS containing 1% BSA, followed by incubation with appropriate horseradish peroxidase (HRP)-conjugated secondary antibodies for 1 h.

Immunoreactive bands were detected using an enhanced chemiluminescence (ECL) kit (GE Healthcare, Chicago, IL, USA), visualized with the ImageQuant™ LAS 4000 system (GE Healthcare, Chicago, IL, USA), and quantified by densitometric analysis using GelQuant.NET freeware (v. 1.8.2). All samples were analyzed in technical duplicates in randomized order. Full images of Western blot membranes are provided in **Supplementary Figure 6**.

### 2.5 Sample preparation for mass spectrometry analysis

High-Select™ Top14 Abundant Protein Depletion Resin (Thermo Scientific, Rockford, IL, USA) was used to remove the 14 most abundant proteins from 10 µL of human plasma in a single step. High-abundance proteins were similarly depleted from murine serum using the Proteome Purify 2 Mouse Serum Protein Immunodepletion Resin (R&D Systems, Minneapolis, MN, USA). The resulting volumes, containing approximately 50 µg of protein after depletion, were then suitably processed for mass spectrometry analyses. Technical triplicates and quality control standards were included for reproducibility.

### 2.6 Liquid chromatography - tandem mass spectrometry analysis

Equal amounts of protein samples (50 µg) were purified by acetone precipitation, using a 1:4 volume ratio (protein sample to ice cold acetone) and incubated at -28 °C for 2 h. The samples were suitably processed for LC–MS/MS analysis as previously described (20). The Easy-nLC II nano-chromatographic system was coupled to the LTQ Orbitrap Velos Pro hybrid mass spectrometer (both from Thermo Scientific, USA) for the analyses. For each sample, 1000 ng of peptides were first trapped in the Acclaim PepMap 100 pre-column (2 cm length × 75 μm inner diameter, 3 µm particle size, 100 Å pore size, Thermo Scientific, USA) and then separated into the Acclaim PepMap 100 analytical column (15 cm length × 75 μm inner diameter, 3 µm particle size, 100 Å pore size, Thermo Scientific, USA) using a 95 min, 6-25% solvent B (99.9% acetonitrile and 0.1% formic acid) over solvent A (99.9% water and 0.1% formic acid) gradient. A 2.0kV direct junction approach was applied to a nano bore stainless-steel emitter (40 mm, O.D. 150 μm, I.D. 30 μm, Thermo Scientific, USA) for peptide nano-electrospray ionization.

The mass spectrometer was operated in a Top 15 data-dependent analysis with 60 k resolution on the full 350–1700 m/z domain, while precursor fragmentation was performed using collision-induced dissociation. Internal calibration was done using the 445.120028 Da polysiloxane peak.

### 2.7 Mass spectrometry data analysis

**Figure 1:**
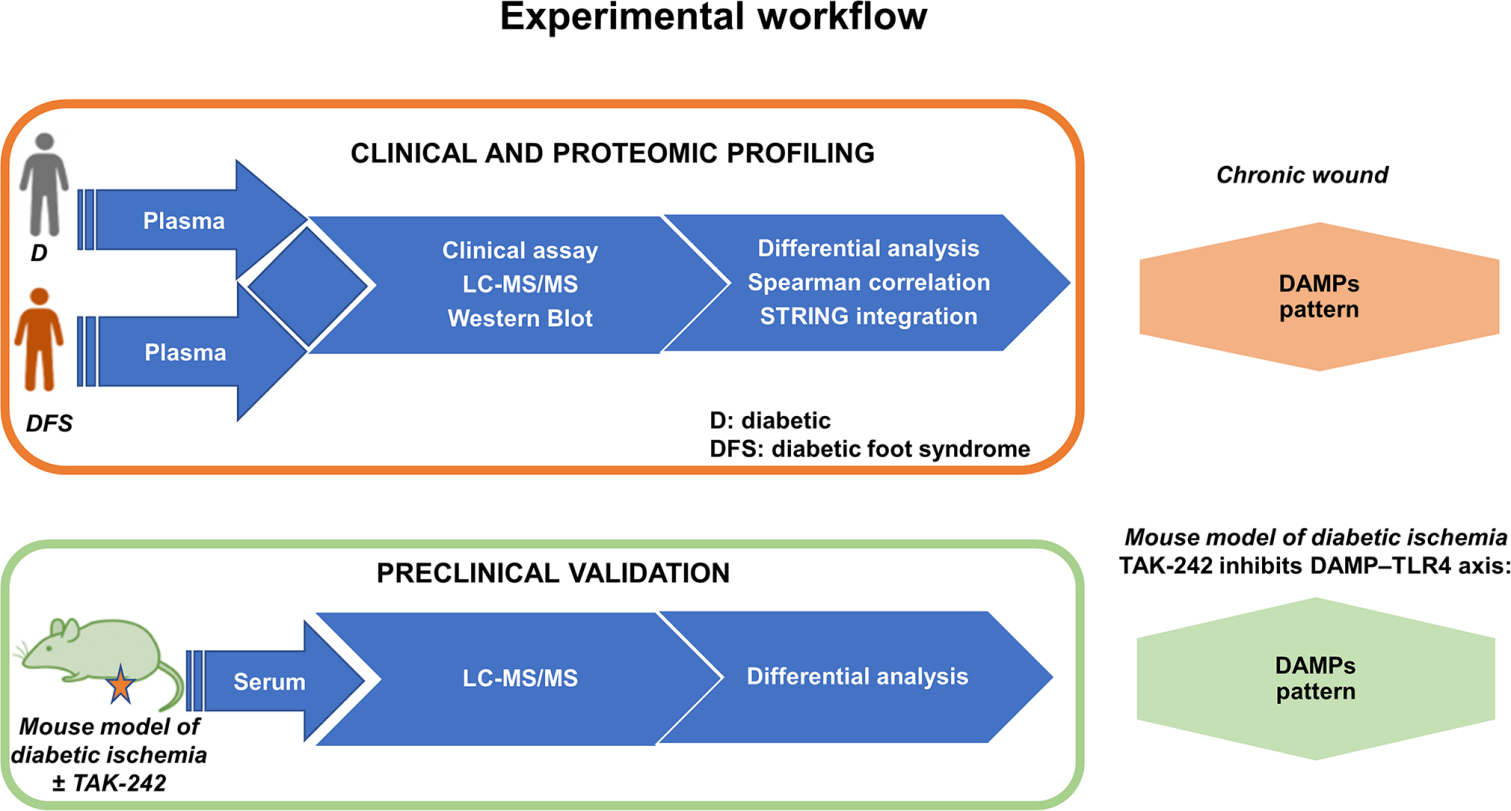
Integrated clinical and experimental workflow. Plasma samples from persons with type 2 diabetes without foot syndrome (D) and patients with diabetic foot syndrome (DFS) were analyzed by LC-MS/MS, Western blotting and Spearman correlation. Preclinical validation was performed in a diabetic ischemic mouse model with or without TLR4 inhibition (TAK-242). D, persons with Type 2 diabetes; DFS, diabetic foot syndrome; DAMPs, Damage-Associated Molecular Patterns.

The raw files were analyzed using Proteome Discoverer 2.4 software (Thermo Scientific). For protein inference, Homo sapiens proteome (TaxID 9606, version 2019-11-04) and Mus musculus proteome (Proteome ID UP000000589, version 04.2019) UniProt/SwissProt reference proteomes were used. Methionine oxidation was set as a dynamic modification, and carbamidomethylation of cysteine was set as a fixed (static) modification. A maximum of two missed tryptic cleavages was permitted. A reverse database search was performed for strict protein and peptide FDR settings (<1%). Label-free relative quantification was performed on the precursor intensity level, and ANOVA hypothesis test enabled. Normalization was engaged in the total peptide amount on controls average. Proteins with a SequestHT score > 10 that were quantified and differentially abundant among the compared groups (> 1.2-fold ratio and p value<0.05) were retained for further analysis. STRING database algorithm (v. 12.0) was used for protein–protein interaction network analysis, integrating evidence from genomic context, high-throughput experiments, automated text mining, and curated databases (21). STRING was also used for gene ontology and KEGG signaling pathways over-representation (**Figure 1**).

### 2.8 Statistical analysis

Results are expressed as mean ± standard deviation (SD). Statistical analyses were performed using GraphPad Prism version 8.0.2. A p-value < 0.05 was considered statistically significant.

Comparisons between two independent groups were performed using the unpaired nonparametric Mann-Whitney U test.

Associations between clinical variables and circulating DAMP abundance were assessed using Spearman’s rank correlation coefficient. Multiple testing correction was performed using the Benjamini-Hochberg false discovery rate (FDR) procedure where appropriate.

For mass spectrometry data, relative spectral abundances of DAMPs were quantified and used for statistical comparisons as described above.

## 3 Results

### 3.1 Clinical characteristics of persons with type 2 diabetes

Baseline demographic and clinical characteristics of the study cohort are summarized in **Table 1**. Patients with DFS and diabetes without foot complications were comparable in terms of age and diabetes duration, while differences were observed in inflammatory and hematological parameters. DFS patients presented more advanced local disease severity, including higher rates of ischemia and surgical interventions.

**Figure 2:**
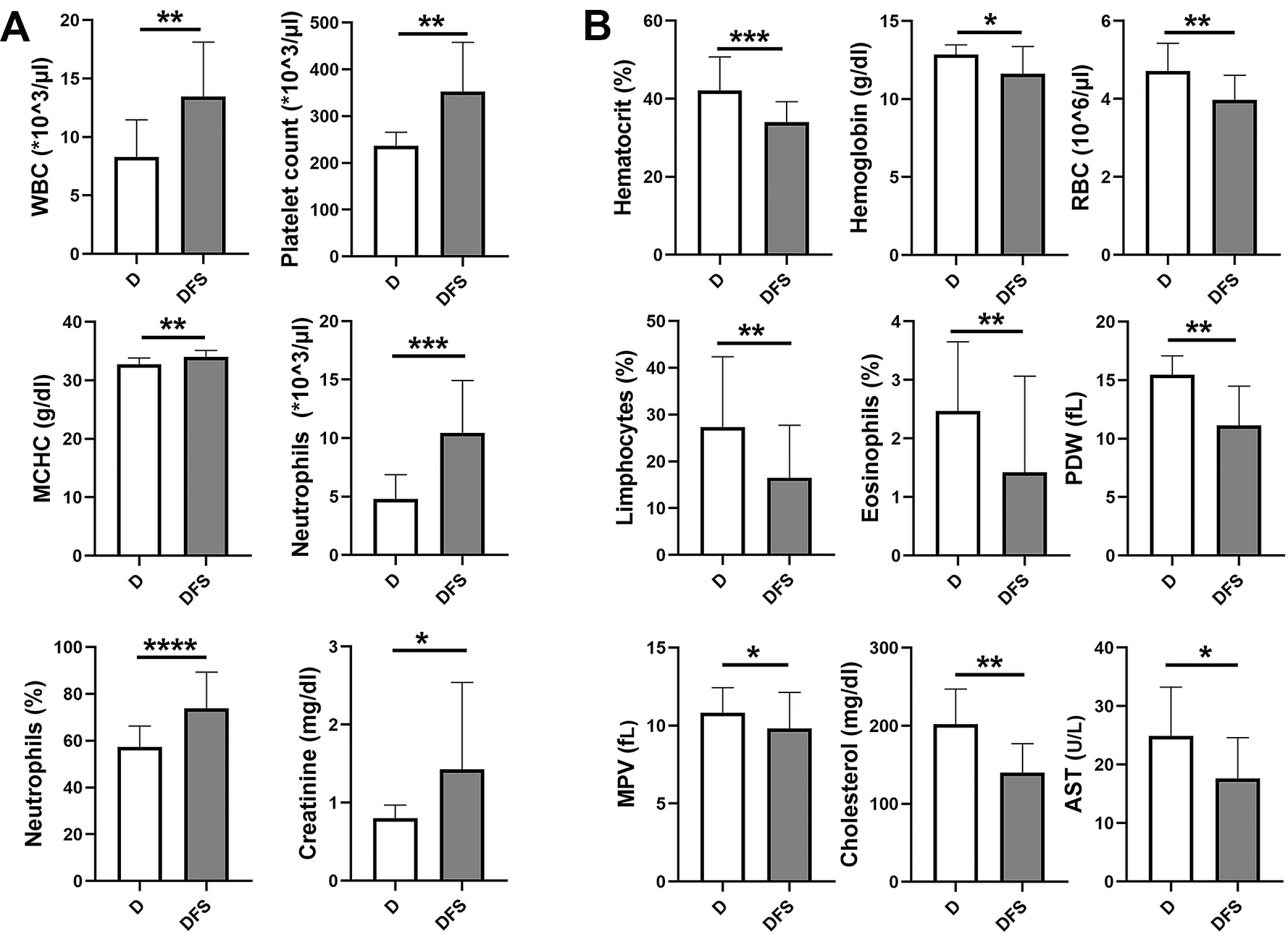
Clinical blood parameter alterations in study groups. **(A)** Patients with diabetic foot syndrome (DFS) exhibited higher counts of leukocytes (WBC), platelets, mean corpuscular hemoglobin concentration (MCHC), and neutrophils compared with persons with Type 2 diabetes without foot complications (D). **(B)** Conversely, DFS patients showed lower levels of hematocrit, hemoglobin, red blood cells (RBC), lymphocytes, eosinophil percentage, platelet distribution width (PDW), mean platelet volume (MPV), cholesterol, and aspartate aminotransferase (AST) compared with D patients (n = 9); DFS: patients (n = 24). D, persons with type 2 diabetes (n = 9); DFS, patients with diabetic foot syndrome (n = 24). Data are presented as mean ± SD. Statistical significance: *P<0.05, **P<0.01, ***P<0.001 vs D.

Clinical and biochemical analyses revealed significant elevations in several blood parameters in patients with DFS compared with the D group (**Figure 2**). The comparative analysis revealed a significant increase in WBC (~1.6-fold; p ≤ 0.01), platelet count (~1.4-fold; p ≤ 0.01), neutrophil count (~2.2-fold; p ≤ 0.001) and creatinine (~1.8-fold, p ≤ 0.05) in DFS vs. D groups (**Figure 2A**). The same comparison revealed a significant reduction in hematocrit (~1.2-fold; p ≤ 0.01), RBC (~1.2-fold; p < 0.01), lymphocyte percentage (~1.7-fold; p ≤ 0.01), eosinophil percentage (~2-fold; p ≤ 0.01), as well as in PDW (~1.3-fold; p ≤ 0.01), total cholesterol (~1.5-fold; p ≤ 0.05) and AST (~1.4-fold; p ≤ 0.05) (**Figure 2B**).

Also, in the DFS group, high values of uric acid, activated partial thromboplastin time (APTT), urea, serum creatinine, leukocytes (WBC), and neutrophils exceeded the reference ranges. Conversely, hematocrit, hemoglobin, lymphocyte percentage, RBC count, and platelet distribution width (PDW) were below the reference ranges.

C-reactive protein (CRP), an inflammation marker, exhibited significantly increased values especially in the DFS (126.15 ± 89.33 mg/dL) but also in D (30.65 ± 32.17 mg/dL) groups, far surpassing the standard limit of <0.5 mg/dL Cholesterol concentrations and mean platelet volume (MPV) exceeded reference ranges in the D group. Additionally, both groups exhibited triglyceride (Tg) levels surpassing the reference ranges. Both the D and DFS groups presented serum glucose values that exceeded reference ranges. These parameters differed significantly between the D and DFS groups.

### 3.2 Comparative shotgun proteomics of persons with type 2 diabetes with and without diabetic foot syndrome

**Figure 3:**
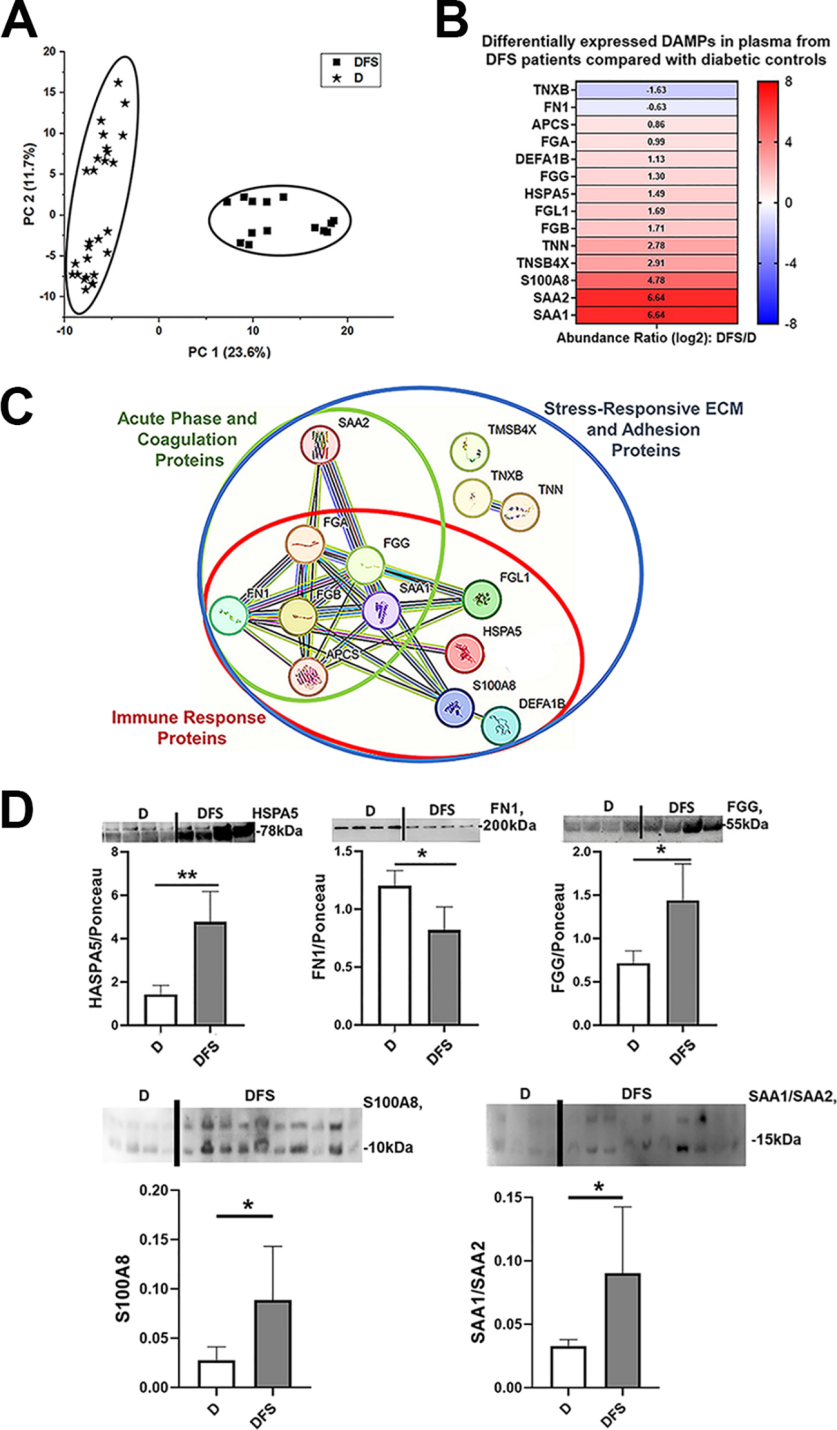
Plasma DAMP profiling and network analysis. **(A)**. Principal component analysis of non-filtered plasma proteomes from diabetic and DFS patients without diabetic foot syndrome **(D)** and patients with diabetic foot syndrome (DFS), showing distinct group separation associated with DFS pathology. **(B)**. Quantitative proteomic analysis of 14 plasma DAMPs (HSPA5; FGA; FGB; FGG; FGL1; S100A8; SAA1; SAA2; TMSB4X; TNXB; TNN; DEFA1B; FN1; APCS). Each plasma sample was analyzed in technical triplicate by LC-MS/MS. **(C)** STRING network-based functional clustering of differentially expressed proteins, identifying three major clusters (1): Acute-phase and coagulation proteins (green; FGA, FGB, FGG, FN1, SAA1, SAA2, APCS) (2); Stress-responsive extracellular matrix and Adhesion-related proteins (blue; HSPA5, FGA, FGB, FGG, FGL1, FN1, DEFA1B, S100A8, SAA1, SAA2, APCS, TNN, TNXB, TMSB4X); and (3) Immune response-associated proteins (red; FGA, FGB, FGG, FN1, SAA1, APCS, DEFA1B, HSPA5, FGL1, S100A8). Node annotations in clusters mean: 78 kDa glucose-regulated protein (HSPA5); Fibrinogen alpha chain (FGA); Fibrinogen beta chain (FGB); Fibrinogen gamma chain (FGG); Fibrinogen-like protein 1 (FGL1); Fibronectin (FN1); Neutrophil defensin 1 (DEFA1B); Protein S100-A8 (S100A8); Serum amyloid A-1 protein (SAA1); Serum amyloid A-2 protein (SAA2); Serum amyloid P-component (APCS); Tenascin-N (TNN); Tenascin-X (TNXB); Thymosin beta-4 (TMSB4X). **(D)** Validation of HSPA5, FN1, FGG, S100A8 and SAA1/SAA2 protein expression by Western blot analysis in plasma samples from all patient groups. Equal amounts of total protein were loaded per lane. HSPA5, FN1 and FGG densitometric signals were normalized to total protein staining (Ponceau S). D: persons with Type 2 diabetes without diabetic foot syndrome (n=9) and DFS: patients with diabetic foot syndrome (n=5). Results are presented as mean ± standard deviation. Statistical significance: * P<0.05, ** P<0.01.

The comparative plasma shotgun proteomic LC-MS experiments led to the unambiguous identification of 406 proteins. Principal component analysis (PCA) revealed a clear separation between D and DFS patients (**Figure 3A**).

PCA showed separation between D and DFS samples. PC1 accounted for 23.6% and PC2 for 11.7% of total variance.

Using a Sequest HT score >10, 397 proteins in D samples, and 379 proteins in DFS group were evidenced. Of the 370 proteins identified across both patient groups, 14 DAMP proteins were differentially abundant in DFS vs. D individuals (see **Supplementary Table 1**). The DFS cohort included patients across multiple Wagner-Armstrong stages with varying ischemic severity. Because of limited subgroup sizes, no statistically powered stratified proteomic analysis according to ulcer severity was performed.

Specifically, as shown in **Figure 3B**, we detected the up-regulation of 78 kDa glucose-regulated protein (∼2.8 fold, p<0.001), Fibrinogen alpha chain (∼2.3 fold, p<0.001), Fibrinogen beta chain (∼3.3 fold, p<0.001), Fibrinogen gamma chain (∼2.4 fold, p<0.001), Fibrinogen-like protein 1 (∼2.9 fold, p<0.05), Protein S100-A8 (∼17.92 fold, p<0.001), Serum amyloid A-1 protein (∼77.7 fold, p<0.001), Serum amyloid A-2 protein (∼80 fold, p<0.001), Serum amyloid P-component (∼1.62 fold, p<0.01), Thymosin beta-4 (∼4.3 fold, p<0.001), Tenascin-N (∼6.2 fold, p<0.001), Neutrophil defensin 1 (∼2.9 fold, p<0.05) and down-regulation of Tenascin-X (∼2.2 fold, p<0.01), Fibronectin (∼1.83 fold, p<0.001) n DFS compared with D.

The bioinformatic analysis of the 14 differentially abundant DAMPs revealed significant over-representation of KEGG pathways and GO categories (see **Supplementary Table 2**). Protein–protein interaction network analysis exposed several functional clusters among differentially expressed proteins (**Figure 3C**). Using STRING-based clustering, we identified three major functional clusters: acute-phase and coagulation proteins (FGA, FGB, FGG, FN1, SAA1, SAA2, APCS), immune response proteins (FGA, FGB, FGG, FN1, SAA1, APCS, DEFA1B, HSPA5, FGL1, S100A8), and stress-responsive extracellular matrix and adhesion proteins (HSPA5, FGA, FGB, FGG, FGL1, FN1, DEFA1B, S100A8, SAA1, SAA2, APCS, TNN, TNXB, TMSB4X).

Immunodepletion may affect proteins associated with carrier complexes. Western blot analysis of non-depleted plasma confirmed increased levels, consistent with LC–MS/MS results. Western blot analysis validated the differential abundance of HSPA5, FGG, FN1, SAA and S100A8 between groups (**Figure 3D**).

### 3.3 Spearman correlation analysis of plasma DAMPs and clinical parameters in DFS patients

**Figure 4:**
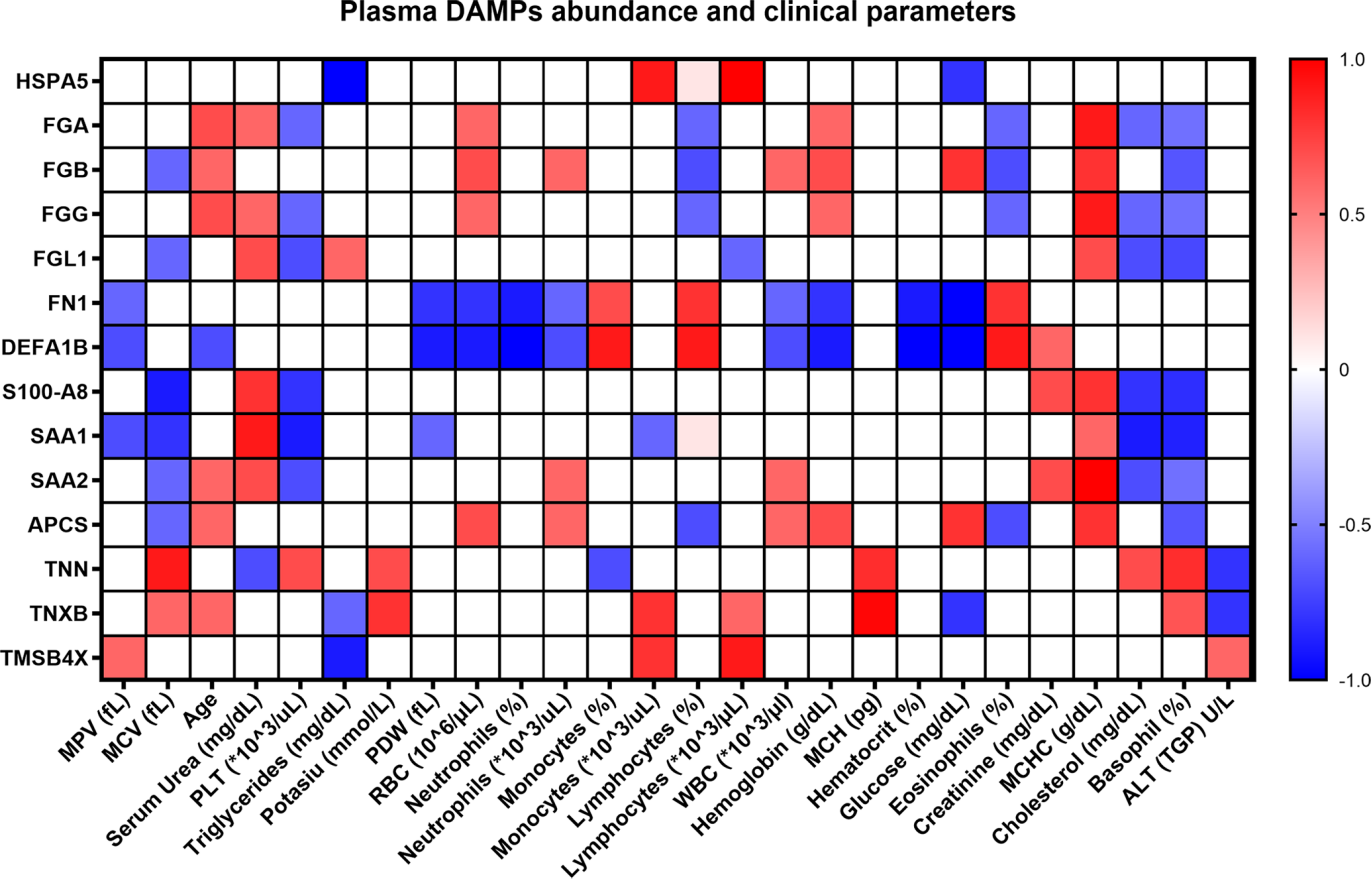
Spearman correlation analysis between plasma DAMPs and clinical parameters in DFS. Spearman rank correlation matrices illustrate monotonic association trends between plasma DAMP abundance and selected clinical parameters in patients with diabetic foot syndrome (DFS). The intensity of red and blue colors indicates the strength of positive and negative correlations, respectively. Correlations with |ρ| ≥ 0.60 are displayed for exploratory visualization purposes.

To explore potential relationships between circulating DAMPs and clinical variables in DFS patients, exploratory Spearman rank correlation analyses were performed (**Figure 4**).

Several correlations (|ρ| ≥ 0.60) were observed between DAMPs and inflammatory, hematological, renal, and metabolic parameters. Triglyceride levels showed an inverse correlation trend with HSPA5/GRP78 abundance, whereas neutrophil percentage demonstrated a negative association with DEFA1B. Lymphocyte counts were positively associated with HSPA5, while hematocrit values showed an inverse association with DEFA1B abundance. In addition, MCHC correlated positively with SAA2, and MCH exhibited a positive association with TNXB.

Additional trends were observed between DAMPs and metabolic or inflammatory variables, including inverse associations of glucose levels with FN1 and TNXB, negative correlations of cholesterol with SAA1, SAA2, and APCS, and positive associations between eosinophil parameters and FN1 abundance. Several hematological indices also demonstrated associations with DAMPs involved in acute-phase signaling and extracellular matrix remodeling. The observed associations should be considered exploratory and interpreted cautiously in the context of the limited cohort size.

### 3.4 Activation of circulating DAMPs via TLR4 in a murine model of diabetic ischemia

**Figure 5:**
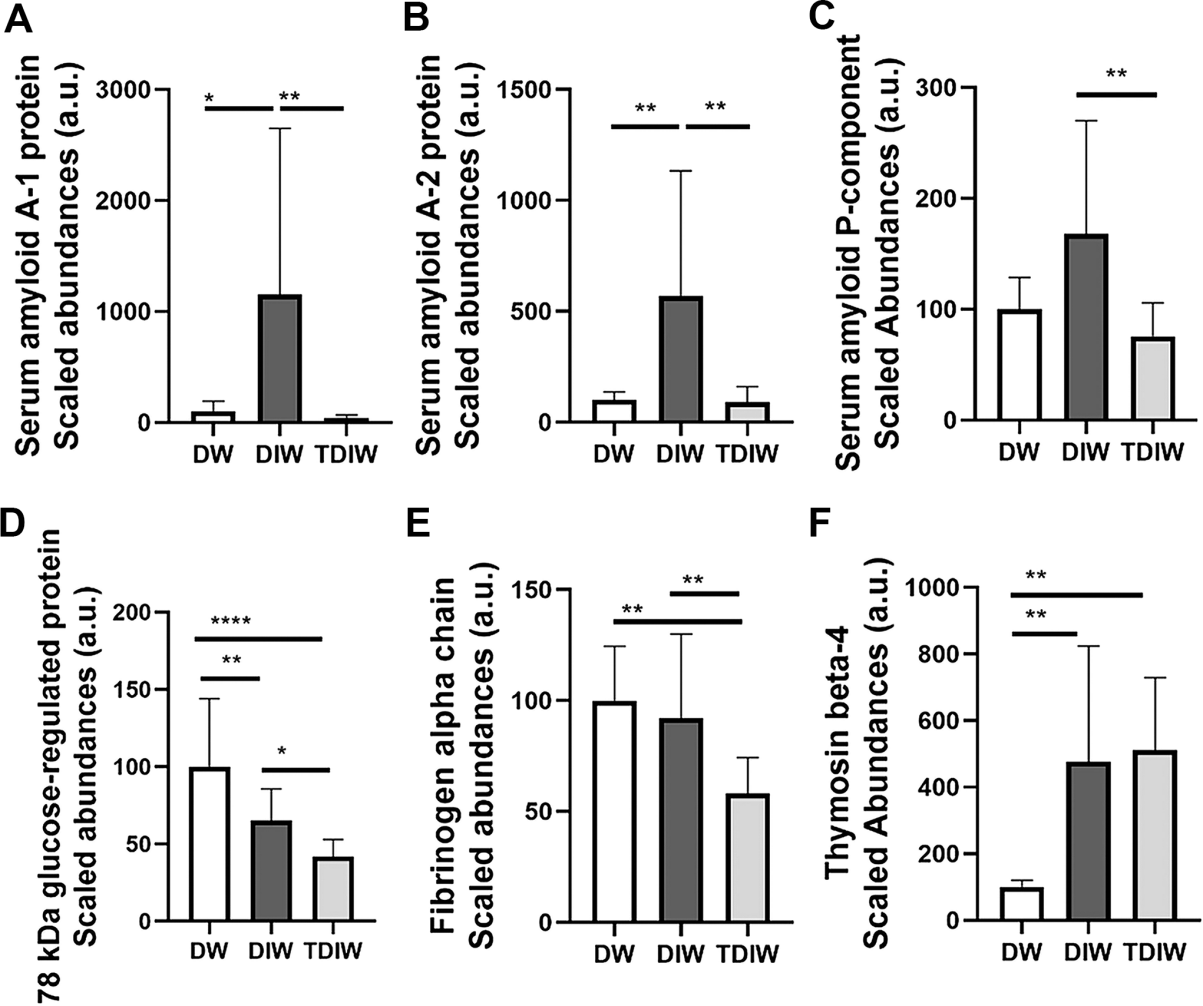
Serum DAMP abundance in a diabetic ischemic wound mouse model. Histograms illustrate significant changes in serum DAMP levels in ischemia and/or TLR4 inhibition: **(A)** serum amyloid A-1 protein, **(B)** serum amyloid A-2 protein, **(C)** serum amyloid P component, **(D)** 78 kDa glucose-regulated protein, **(E)** fibrinogen alpha chain and **(F)** thymosin beta-4. Each serum sample was analyzed in technical triplicate by liquid chromatography-tandem mass spectrometry (LC-MS/MS). Experimental groups included DW (diabetic wound, n=3); DIW (diabetic ischemic wound, n=6), and TDIW (DIW treated with TAK-242, n=5). Data are presented as mean ± SD. Statistical significance: *P<0.05, **P<0.01, ****P<0.0001.

To explore the signaling pathways and therapeutic potential of the DAMPs identified in the clinical cohort, we employed a murine model of streptozotocin-induced diabetes with plantar wounds, with and without arterial ischemia. Blood glucose levels and body weight were monitored during the experimental protocol to confirm the diabetic phenotype of the animals. Mean blood glucose values were 488.5 ± 156.1 mg/dL in DW mice, 512.6 ± 119.1 mg/dL in DIW mice, and 456.7 ± 82.7 mg/dL in TDIW mice. Mean body weight was 16.3 ± 3.1 g in DW mice, 16.6 ± 1.4 g in DIW mice, and 22.3 ± 2.2 g in TDIW mice. The pharmacological effects of TLR4 inhibition, using TAK-242 agent, were tested. Proteomic analysis of murine sera revealed significant effects on the expression of DAMP proteins after TLR4 inhibition. SAA1, SAA2, and APCS abundances were reduced in TDIW compared to DIW (**Figures 5A–C**).

In contrast, HSPA5, FGA, and TMSB4X showed significant changes that appeared to amplify, rather than counteract, the alterations induced by DIW (**Figures 5D–F**).

## 4 Discussion

The present integrative study combines clinical and biochemical evaluations with LC-MS/MS plasma proteomics to define systemic DAMP- related inflammatory signaling potentially involving TLR4 in diabetic foot syndrome and to explore its pharmacological modulation. Our findings suggest the presence of a systemic molecular pattern associated with DFS marked by dysregulated DAMPs involved in inflammation, coagulation, and extracellular matrix (ECM) remodeling, distinguishing DFS patients from diabetics without foot complications. Increased plasma abundance of fibrinogen subunits (FGA, FGB, FGG), acute-phase proteins (SAA1, SAA2, CRP), stress-related proteins (HSPA5), and innate immune mediators (S100A8, DEFA1B), together with a concomitant reduction in key ECM-associated DAMPs (FN1, TNXB) and altered levels of matricellular proteins (TNN, TMSB4X), were observed in DFS. Correlation trends between several DAMPs and clinical or metabolic parameters were observed; however, these associations did not remain significant after multiple-testing correction and should therefore be interpreted cautiously. Nevertheless, the overall proteomic pattern is consistent with the possibility that sterile, DAMP-driven inflammation may be coupled to ECM destabilization and impaired tissue repair in DFS. Pharmacological interrogation in a streptozotocin-induced diabetic ischemia model showed selective modulation of several acute-phase DAMPs following TLR4 inhibition, providing preliminary observations consistent with a possible involvement of TLR4 signaling in systemic inflammatory regulation. Although we employed a type 1 diabetes murine model, these findings suggest a potential role of TLR4-driven inflammatory processes in diabetic ischemic injury. It should be noted that the experimental model represents streptozotocin-induced diabetes and therefore does not entirely mimic the clinical setting of type 2 diabetic foot syndrome. The inflammatory and metabolic milieu of streptozotocin-induced diabetes differs substantially from type 2 diabetes, including differences in insulin resistance, adipose tissue inflammation, and immune regulation, which may influence DAMP–TLR4 signaling. Therefore, the murine findings should be interpreted as mechanistic observations rather than direct recapitulation of human DFS pathophysiology.

A major strength of this study lies in its integrative design, which combines unbiased plasma proteomics with routine clinical parameters and correlation analyses, enabling an integrated exploratory assessment of DFS pathophysiology at the systemic level. The integration of proteomic findings with clinical markers of thrombosis and inflammation, such as platelet indices, CRP, leukocyte counts, and renal parameters, is broadly consistent with the potential biological relevance of the identified DAMP signature. Moreover, the inclusion of a preclinical murine model of diabetic ischemia treated with a TLR4 inhibitor provides preliminary mechanistic evidence for the involvement of DAMP-mediated inflammatory pathways, reinforcing the translational relevance of the human data.

Several limitations should be considered. The cross-sectional design precludes causal inference and longitudinal assessment of disease progression or prognostic value. Moreover, comorbid conditions commonly present in DFS, such as renal dysfunction and metabolic syndrome, may influence circulating protein levels and cannot be fully disentangled in the present analysis. The absence of a healthy non-diabetic control group may further limit conclusions regarding disease specificity.

Finally, differences in biological matrices between species (human plasma versus murine serum) may influence the interpretation of coagulation- and inflammation-related proteins and limit direct quantitative comparisons between human and experimental datasets.

Previous studies have demonstrated that fibrinogen, serum amyloid proteins, CRP, and ECM components contribute to vascular inflammation and tissue injury in diabetes, sepsis, and other sterile inflammatory conditions (22–25). However, most investigations have focused on isolated mediators or tissue-specific alterations rather than integrated circulating molecular networks. In contrast, our plasma-based proteomic profiling reveals a coordinated DAMP signature in human DFS, linking inflammation, coagulation, and ECM remodeling within a single systemic framework. Nevertheless, given the limited cohort size and class imbalance, these findings should be interpreted cautiously and viewed as hypothesis-generating rather than evidence of immediate biomarker applicability. Independent validation in larger cohorts will be required to determine their potential diagnostic or prognostic relevance.

While DAMPs such as SAA1, FGB, FGG, and CRP, as well as ECM-associated molecules including FN1, have been implicated in vascular inflammation and sterile injury models (24), correlation trends involving eosinophil- and basophil-related parameters were observed in the present cohort. Similarly, neutrophil activation and defensin release have been extensively studied in sepsis and acute vascular injury (26, 27). While these studies primarily focus on acute lung or systemic inflammation, the relevance of neutrophil-derived defensins in chronic diabetic complications has remained unexplored. Our findings suggest that similar mechanisms may also operate to diabetic foot pathology, highlighting neutrophil-derived defensins and innate immune activation as contributors to chronic inflammation and tissue damage in this setting.

Furthermore, whereas previous studies have linked circulating HSPA5 primarily to obesity and type 2 diabetes (28), our data reveal increased plasma HSPA5 specifically in DFS, indicating enhanced extracellular release under ER stress in the context of chronic foot lesions. Altered expression of ECM-related proteins, including upregulation of TNN and downregulation of TNXB, aligns with prior observations that tenascins are differentially regulated by microenvironmental cues (29), while extending the concept of ECM dysregulation beyond cardiac tissue to plasma-detectable systemic alterations (30).

The molecular patterns identified in this study are consistent with a model in which persistent sterile inflammation in DFS is associated with systemic DAMP dysregulation and TLR4-dependent signaling, linking metabolic stress, coagulation activation, and extracellular matrix remodeling within a coordinated pathogenic network. Elevated fibrinogen chains and platelet-related alterations indicate a pro-thrombotic milieu, while reduced circulating fibronectin may reflect excessive tissue deposition or impaired turnover, consistent with maladaptive ECM remodeling in chronic inflammation (31). Increased TMSB4X levels may represent a compensatory angiogenic response or matricellular response (32, 33), whereas correlation trends between DAMPs and metabolic parameters may be consistent with previously reported effects of hyperglycemia and dyslipidemia on vascular injury (34). Although direct activation of TLR4 was not measured, the pharmacological feedback of some DAMPs to TAK-242 provides preliminary experimental observations consistent with TLR4 critical role in systemic inflammatory regulation in DFS.

Notably, the differential responsiveness of DAMP subsets to TLR4 inhibition suggests the existence of pharmacologically distinct inflammatory modules. Acute-phase amyloid proteins exhibited TLR4-dependent regulation, whereas stress-response and matrix-associated molecules appeared to be modulated through parallel or compensatory pathways. This heterogeneity may have therapeutic implications, indicating that selective TLR4 inhibition alone may not fully normalize systemic DAMP dysregulation in DFS and that multi-target strategies may be required.

Clinically, the identification of a circulating DAMP-associated profile linked to DFS relative to diabetic controls without foot complications has potential implications for patient stratification, disease progression monitoring, and evaluation of therapeutic responses. Mechanistically anchored plasma DAMP signatures may serve as pharmacodynamic indicators of inflammatory pathway activation and therapeutic response in DFS. From a broader perspective, the upstream targeting of DAMP-TLR4 signaling pathways may offer novel therapeutic opportunities aimed at modulating sterile inflammation rather than addressing downstream consequences alone, potentially reducing ulcer progression, amputations, and healthcare burden.

It remains unclear whether the identified DAMPs actively drive DFS progression or primarily reflect established tissue damage. Longitudinal studies are required to determine their predictive value for ulcer severity, healing outcomes, and amputation risk. Further investigation is also needed to clarify tissue-plasma relationships and to assess how local wound microenvironments contribute to systemic DAMP release.

Future research should incorporate larger, multicenter cohorts and multi-omics approaches to refine the DFS molecular signature and identify upstream regulatory mechanisms. Interventional studies targeting DAMP–TLR4 signaling in humans, building on the translational evidence provided by TAK-242 in experimental diabetic ischemia, will be essential for translating these findings into clinically actionable strategies.

In conclusion, this work suggests that systemic DAMP dysregulation may represent a feature of diabetic foot syndrome and provides functional evidence supporting TLR4-dependent regulation of acute-phase inflammatory responses. By integrating clinical molecular profiling with experimental pharmacology, this study provides a preliminary mechanistic framework for hypothesis-driven biomarker development and targeted immunomodulatory strategies in diabetic ischemic complications. While the DAMP-TLR4 axis emerges as a candidate pharmacologically relevant pathway, further validation in larger cohorts and human-focused mechanistic studies will be required for clinical translation.

## Data Availability

The mass spectrometry proteomics data have been deposited to the ProteomeXchange Consortium via the PRIDE (35, 36) partner repository with the dataset identifier PXD073507 and 10.6019/PXD073507.

## Supplementary material

Supplementary Table 1 List of DAMP molecules exhibiting a statistically significant abundance alteration of at least 1.2-fold in the plasma collected from DFS patients.

Supplementary Table 2 Functional clustering of differentially expressed plasma proteins identified in study participants, based on STRING network analysis.
