# Supplementary material for: Dysregulation of circulating damage-associated molecular patterns in diabetic foot syndrome
Source: Front Immunol. 2026 Jun 22;17:1849387. doi: 10.3389/fimmu.2026.1849387 (PMC13333410; doi:10.3389/fimmu.2026.1849387)
Supplement: Supplementary Table 1 — List of DAMP molecules exhibiting a statistically significant abundance alteration of at least 1.2-fold in the plasma collected from DFS patients. [file DataSheet1.docx]

Supplementary Material

**Dysregulation of circulating damage-associated molecular patterns in diabetic foot syndrome**

**Elena Uyy^1#^, Viorel‑Iulian Suica^1#^, Luminita Ivan^1^, Raluca Maria Boteanu^1^, Valentina Diana Uta^1^, Elena Georgiana Bernea^1^, Dragoș Eugen Georgescu^2^, Ovidiu Chiriac^2^, Maya Simionescu^1^, Felicia Antohe^1, *^**

**^1^**Institute of Cellular Biology and Pathology “Nicolae Simionescu”, Bucharest, Romania

**^2”^** Dr I Cantacuzino” Hospital, Bucharest, Romania

*Corresponding Author: Felicia Antohe

Postal address: Proteomics Department, Institute of Cellular Biology and Pathology “Nicolae Simionescu”, 8, B.P. Hasdeu Street, PO Box 35–14, 050568, Bucharest, Romania

Phone : +40 21 3192737 ;

Email : felicia.antohe@icbp.ro

#Elena Uyy, Viorel‑Iulian Suica have contributed equally to this work.

**Supplemental Table S1**:

List of DAMP molecules exhibiting a statistically significant abundance alteration of at least 1.2-fold in the plasma collected from DFS patients. The Uniprot accession code, Sequest Score, and the number of unique peptides for each molecule were included in the table.

| **No.** | **Uniprot Accession** | **Protein description** | | **Gene Symbols** | | **Unique Peptides** | | **Sequest HT Score** | |
| --- | --- | --- | --- | --- | --- | --- | --- | --- | --- |
| 1 | P11021 | 78 kDa glucose-regulated protein | HSPA5 | | 3 | | 64.99 | |  |
| 2 | P02671-1 | Fibrinogen alpha chain | FGA | | 42 | | 16537.71 | |  |
| 3 | P02675 | Fibrinogen beta chain | FGB | | 45 | | 16832.72 | |  |
| 4 | P02679 | Fibrinogen gamma chain | FGG | | 27 | | 10816.44 | |  |
| 5 | Q08830 | Fibrinogen-like protein 1 | FGL1 | | 5 | | 58.16 | |  |
| 6 | P02751 | Fibronectin | FN1 | | 113 | | 44264.89 | |  |
| 7 | P59665 | Neutrophil defensin 1 | DEFA1B | | 3 | | 72.82 | |  |
| 8 | P05109 | Protein S100-A8 | S100A8 | | 5 | | 101.2 | |  |
| 9 | P0DJI8 | Serum amyloid A-1 protein | SAA1 | | 7 | | 6205.54 | |  |
| 10 | P0DJI9 | Serum amyloid A-2 protein | SAA2 | | 6 | | 3159.57 | |  |
| 11 | P02743 | Serum amyloid P-component | APCS | | 12 | | 4144.16 | |  |
| 12 | Q9UQP3 | Tenascin-N | TNN | | 1 | | 49.86 | |  |
| 13 | P22105 | Tenascin-X | TNXB | | 7 | | 43.96 | |  |
| 14 | P62328 | Thymosin beta-4 | TMSB4X | | 3 | | 401.58 | |  |

**Supplemental Table S2:**

Functional clustering of differentially expressed plasma proteins identified in study participants, based on STRING network analysis. The interaction map groups query proteins into three main clusters: (1) Acute phase and coagulation proteins (green) include coagulation and acute-phase proteins (FGA, FGB, FGG, FN1, SAA1, SAA2, APCS); (2) Stress-responsive ECM and Adhesion proteins (blue) involve ECM components and stress-response proteins (HSPA5, FGA, FGB, FGG, FGL1, FN1, DEFA1B, S100A8, SAA1, SAA2, APCS, TNN, TNXB, TNSB4X); and (3) Immunes response proteins (red), a cluster linked to immune activation (FGA, FGB, FGG, FN1, SAA1, APCS, DEFA1B, HSPA5, FGL1, S100A8). Node annotations in clusters represent:78 kDa glucose-regulated protein (HSPA5); Fibrinogen alpha chain (FGA); Fibrinogen beta chain (FGB); Fibrinogen gamma chain (FGG); Fibrinogen-like protein 1 (FGL1); Fibronectin (FN1); Neutrophil defensin 1 (DEFA1B); Protein S100-A8 (S100A8); Serum amyloid A-1 protein (SAA1); Serum amyloid A-2 protein (SAA2); Serum amyloid P-component (APCS); Tenascin-N (TNN); Tenascin-X (TNXB); Thymosin beta-4 (TNSB4X). Colour coding of STRING clusters: Cluster 1 (green): Acute phase and coagulation proteins; Cluster 2 (blue): Stress-responsive ECM and adhesion proteins; Cluster 3 (red): Immune response proteins

| **#Category** | **Term ID** | **Term description** | **Observed gene count** | **Background gene count** | **Strength** | **Signal** | **False discovery rate** | **Matching proteins in network (labels)** |
| --- | --- | --- | --- | --- | --- | --- | --- | --- |
| WikiPathways | WP4927 | COVID-19, thrombosis and anticoagulation | 3 | 7 | 2.75 | 2.51 | 8.32E-06 | FGB,FGG,FGA |
| GO Process | GO:0072378 | Blood coagulation, fibrin clot formation | 4 | 24 | 2.34 | 2.43 | 7.56E-06 | FGB,FGG,FN1,FGA |
| UniProt Keywords | KW-0011 | Acute phase | 3 | 19 | 2.32 | 1.94 | 9.14E-05 | FN1,SAA1,SAA2 |
| GO Process | GO:0006953 | Acute-phase response | 4 | 42 | 2.1 | 2.04 | 3.68E-05 | APCS,FN1,SAA1,SAA2 |
| UniProt Keywords | KW-0094 | Blood coagulation | 3 | 46 | 1.93 | 1.44 | 0.00072 | FGB,FGG,FGA |
| STRING clusters | CL:18726 | Complement and coagulation cascades, and Protein-lipid complex | 6 | 161 | 1.69 | 2.02 | 7.26E-06 | APCS,FGB,FGG,SAA1,SAA2,FGA |
| STRING clusters | CL:18728 | Complement and coagulation cascades, and Positive regulation of opsonization | 4 | 109 | 1.68 | 1.34 | 0.00074 | APCS,FGB,FGG,FGA |
| KEGG | hsa04610 | Complement and coagulation cascades | 3 | 82 | 1.68 | 0.88 | 0.0114 | FGB,FGG,FGA |
| GO Component | GO:0062023 | Collagen-containing extracellular matrix | 10 | 407 | 1.51 | 2.74 | 9.01E-11 | TNN,APCS,FGB,FGG,FN1,S100A8,DEFA1B,FGL1,TNXB,FGA |
| Reactome | HSA-1474244 | Extracellular matrix organization | 6 | 300 | 1.42 | 1.72 | 5.41E-06 | TNN,FGB,FGG,FN1,TNXB,FGA |
| GO Process | GO:0007160 | Cell-matrix adhesion | 6 | 136 | 1.76 | 2.21 | 3.12E-06 | TNN,FGB,FGG,FN1,TNXB,FGA |
| GO Function | GO:0005201 | Extracellular matrix structural constituent | 5 | 131 | 1.7 | 1.66 | 0.0001 | FGB,FGG,FN1,TNXB,FGA |
| GO Process | GO:0080134 | Regulation of response to stress | 9 | 1373 | 0.93 | 0.88 | 0.00012 | APCS,FGB,HSPA5,FGG,S100A8,TMSB4X,SAA1,FGA |
| GO Process | GO:0006950 | Response to stress | 11 | 3358 | 0.63 | 0.51 | 0.0012 | APCS,FGB,HSPA5,FGG,FN1,S100A8,DEFA1B,SAA1,SAA2,FGA |
| Reactome | HSA-1280218 | Adaptive Immune System | 6 | 758 | 1.02 | 0.87 | 0.00074 | FGB,HSPA5,FGG,S100A8, FGA |
| Reactome | HSA-168249 | Innate Immune System | 7 | 1041 | 0.95 | 0.84 | 0.00033 | FGB,FGG,S100A8,DEFA1B,SAA1,FGA |
| GO Process | GO:0045087 | Innate immune response | 5 | 754 | 0.94 | 0.54 | 0.0244 | APCS,FGB,S100A8,DEFA1B,FGA |
| GO Process | GO:0006955 | Immune response | 6 | 1321 | 0.78 | 0.44 | 0.0332 | APCS,FGB,S100A8,DEFA1B,FGL1,FGA |
| Reactome | HSA-168256 | Immune System | 9 | 1979 | 0.78 | 0.69 | 0.0002 | FGB,HSPA5,FGG,FN1,S100A8,DEFA1B,SAA1,FGA |
| GO Process | GO:0002376 | Immune system process | 8 | 2121 | 0.69 | 0.48 | 0.0098 | APCS,FGB,S100A8,DEFA1B,FGL1,SAA1,FGA |

**Supplemental Figure S6**. Full images of Western blot membranes


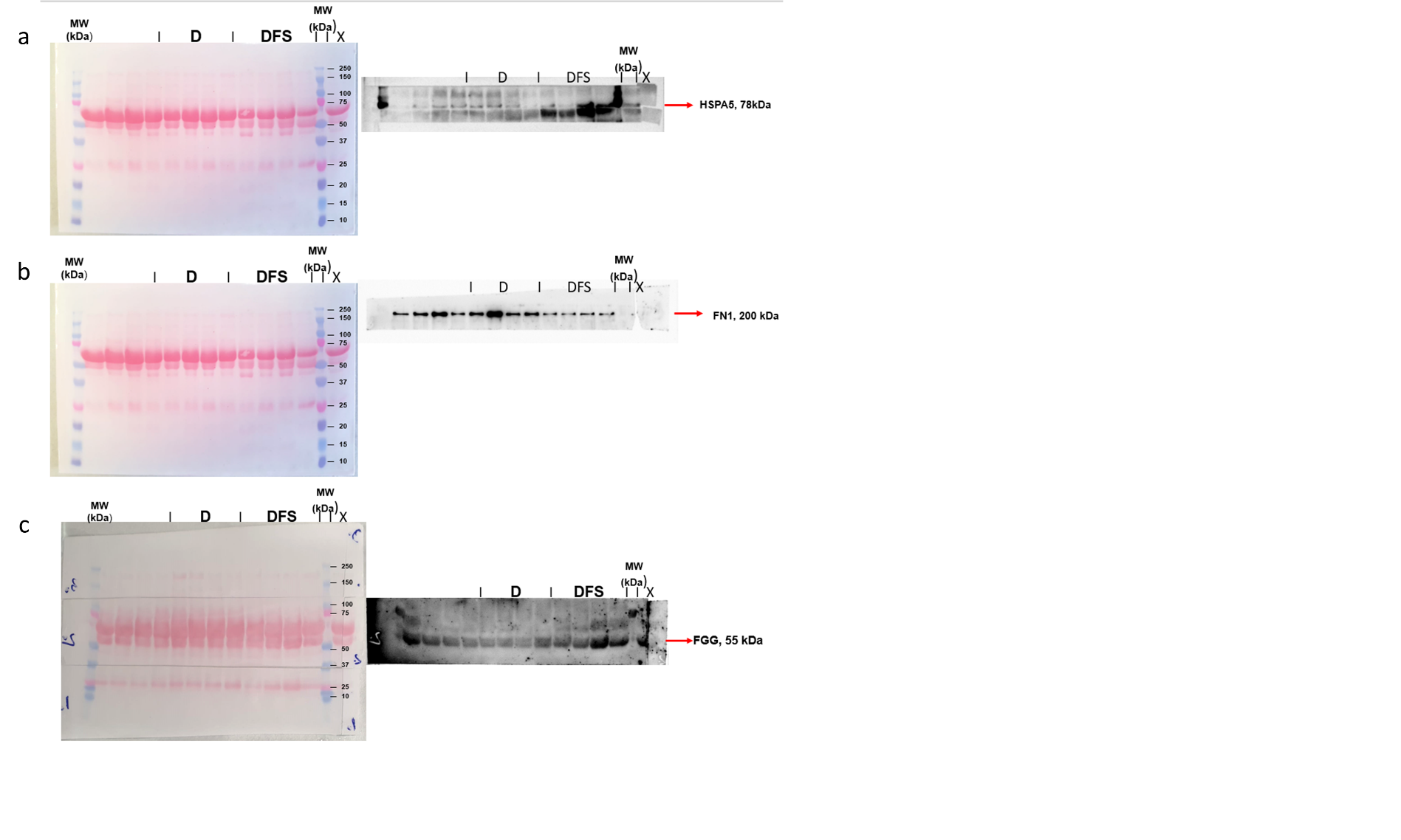


**Supplemental Figure S6**.

Full-length blots of the images presented in Figure 2, which were used for the detection of: a) HSPA5 (Ab21685), b) fibronectin (Ab6328), and c) Fibrinogen gamma (MA5-47028) proteins in plasma samples from all patient groups included in the study. Membranes were stained with Ponceau S to visualize total protein prior to blocking and incubating with the primary antibodies; this staining was also used to normalize of the HSPA5, FN1, and FGG proteins levels. For Sample X, a single loaded plasma lane was vertically cut into two halves: the left half served as a positive control with primary antibody 1, while the right half was incubated only with primary antibody 2 to confirm specificity and the absence of non-specific binding.
